# Supplementary figures and images for: A modified CD9 tag for efficient protein delivery via extracellular vesicles
Source: PLoS One. 2024 Oct 17;19(10):e0310083. doi: 10.1371/journal.pone.0310083 (PMC11486436; doi:10.1371/journal.pone.0310083)

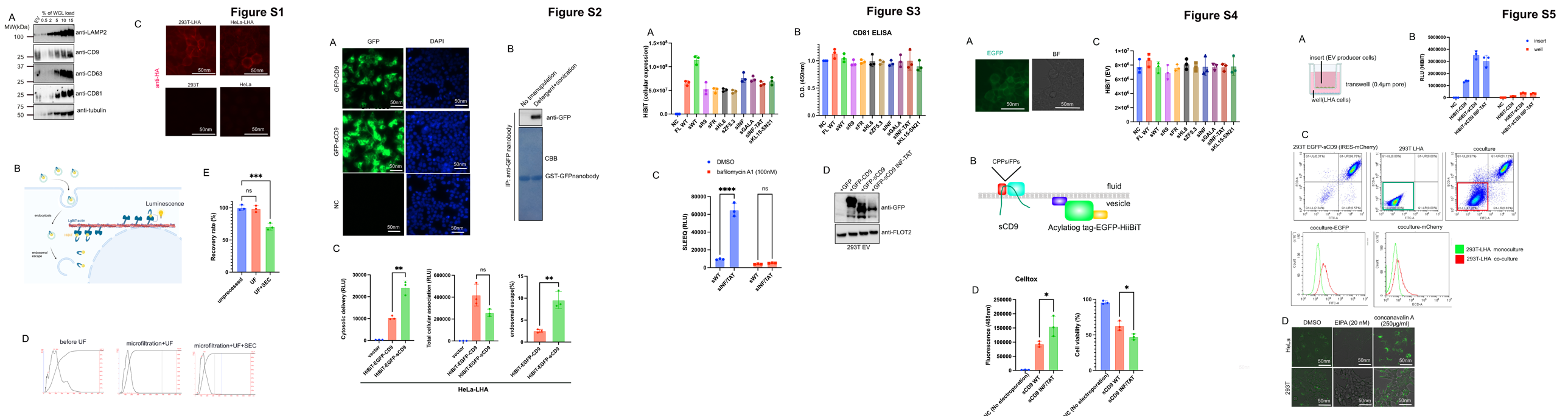

Supplement: S1 File — (JPG) [file pone.0310083.s001.jpg]
